# Study protocol for a phase 2A trial of the safety and tolerability of increased dose rifampicin and adjunctive linezolid, with or without aspirin, for HIV-associated tuberculous meningitis [LASER-TBM]

**DOI:** 10.12688/wellcomeopenres.16783.1

**Published:** 2021-06-01

**Authors:** Angharad G. Davis, Sean Wasserman, Mpumi Maxebengula, Cari Stek, Marise Bremer, Remy Daroowala, Saalikha Aziz, Rene Goliath, Stephani Stegmann, Sonya Koekemoer, Amanda Jackson, Louise Lai Sai, Yakub Kadernani, Thandi Sihoyiya, C.Jason Liang, Lori Dodd, Paolo Denti, Thomas Crede, Jonathan Naude, Patryk Szymanski, Yakoob Vallie, Ismail Banderker, Shiraz Moosa, Peter Raubenheimer, Rachel P.J. Lai, John Joska, Sam Nightingale, Anna Dreyer, Gerda Wahl, Curtis Offiah, Isak Vorster, Sally Candy, Frances Robertson, Ernesta Meintjes, Gary Maartens, John Black, Graeme Meintjes, Robert J. Wilkinson

**Affiliations:** 1 The Francis Crick Institute, Midland Rd, London, NW1 1AT, UK; 2 Faculty of Life Sciences, University College London, London, WC1E 6BT, UK; 3 Wellcome Centre for Infectious Diseases Research in Africa, University of Cape Town, Observatory, Cape Town, 7925, South Africa; 4 Department of Medicine, University of Cape Town, Observatory, 7925, South Africa; 5 Department of Infectious Diseases, Imperial College London, London, W12 0NN, UK; 6 Biostatistics Research Branch, National Institute of Allergy and Infectious Diseases, Maryland, USA; 7 Division of Clinical Pharmacology, Department of Medicine, University of Cape Town, Observatory, 7925, South Africa; 8 Mitchells Plain Hospital, 8 A Z Berman Drive, Lentegeur, Cape Town, 7785, South Africa; 9 New Somerset Hospital, Portswood Rd, Green Point, Cape Town, 8051, South Africa; 10 Department of Psychiatry and Mental Health, HIV Mental Health Research Unit, Neuroscience Institute, University of Cape Town, Observatory, 7925, South Africa; 11 Department of Medicine, Water Sisulu University, Mthatha, 5117, South Africa; 12 Department of Neuroradiology, Imaging Department, Royal London Hospital, Barts Health NHS Trust, London, E1 1BB, UK; 13 Division of Diagnostic Radiology, University of Cape Town, Groote Schuur Hospital, Observatory, 7925, South Africa; 14 MRC/UCT Medical Imaging Research Unit Department of Human Biology, Faculty of Health Sciences, University of Cape Town, Observatory, 7925, South Africa

**Keywords:** Tuberculous meningitis, HIV, Rifampicin, Aspirin, Linezolid

## Abstract

**Background:** Tuberculous meningitis (TBM) is the most lethal form of tuberculosis with a mortality of ~50% in those co-infected with HIV-1. Current antibiotic regimens are based on those known to be effective in pulmonary TB and do not account for the differing ability of the drugs to penetrate the central nervous system (CNS). The host immune response drives pathology in TBM, yet effective host-directed therapies are scarce. There is sufficient data to suggest that higher doses of rifampicin (RIF), additional linezolid (LZD) and adjunctive aspirin (ASA) will be beneficial in TBM yet rigorous investigation of the safety of these interventions in the context of HIV associated TBM is required. We hypothesise that increased dose RIF, LZD and ASA used in combination and in addition to standard of care for the first 56 days of treatment with be safe and tolerated in HIV-1 infected people with TBM.

**Methods:** In an open-label randomised parallel study, up to 100 participants will receive either; i) standard of care (n=40, control arm), ii) standard of care plus increased dose RIF (35mg/kg) and LZD (1200mg OD for 28 days, 600mg OD for 28 days) (n=30, experimental arm 1), or iii) as per experimental arm 1 plus additional ASA 1000mg OD (n=30, experimental arm 2). After 56 days participants will continue standard treatment as per national guidelines. The primary endpoint is death and the occurrence of solicited treatment-related adverse events at 56 days. In a planned pharmacokinetic (PK) sub-study we aim to assess PK/pharmacodynamic (PD) of oral vs IV rifampicin, describe LZD and RIF PK and cerebrospinal fluid concentrations, explore PK/PD relationships, and investigate drug-drug interactions between LZD and RIF. Safety and pharmacokinetic data from this study will inform a planned phase III study of intensified therapy in TBM.

**Clinicaltrials.gov registration: **NCT03927313 (25/04/2019)

## Introduction

In Africa HIV-1 associated tuberculous meningitis (TBM) has a 2-month mortality approaching 50% ^1^. Although early antiretroviral therapy (ART) is of proven benefit in other forms of tuberculosis (TB) ^2^ this has not been demonstrated for TBM ^3^, a finding potentially contributed to by exacerbated immunopathology in the confined space of the central nervous system (CNS) ^4^. Furthermore only a few clinical trials have addressed the recognized poor penetration of several antitubercular agents into cerebrospinal fluid (CSF) and adjunctive corticosteroids have not shown unequivocal benefit for HIV-1 co-infected patients in clinical trials ^5^. The recommended management of patients with TBM has remained unchanged for decades. The need to develop an effective drug treatment regimen combining agents to ensure effective bacterial killing, as well as therapies to control the host immune response is urgent.

### Linezolid

Linezolid (LZD) is currently used as an effective rescue therapy in extensively drug resistant TB ^6– 11^. Its use is also established for the treatment of gram positive infections including pyogenic brain abscesses where patients receive 1200mg for four weeks ^12^. LZD is an attractive agent for the treatment of TBM due to its potent efficacy against *Mycobacterium tuberculosis (M.tb)* as well as its excellent CNS penetration ^12^. Two published studies have investigated its potential role in TBM; the first, an observational study demonstrated favorable clinical outcomes and a non-significant difference in adverse events in children with drug sensitive TBM treated with LZD compared to control ^13^; the second, a retrospective cohort study of 33 adults with TBM found that the addition of LZD to a standard regimen led to more rapid improvement in CSF parameters, recovery of consciousness and reduction of fever ^14^.

LZD toxicity has however limited its use. The most common adverse events (AE) associated with LZD use in TB treatment are haematological toxicity (mainly dose-related) and peripheral neuropathy (mainly duration-dependent) ^15^. These are usually mild and are reversible with dose reduction or treatment interruption if identified early. In a systematic review, AE related to LZD use at doses > 600 mg/day occurred at a median of 252 days (IQR 120 – 540) of LZD exposure ^16^. In the *NiX-TB* trial ^17^ where LZD was given at a total dose of 1200mg per day for 6 months, peripheral neuropathy occurred in 81% of cases with the majority of these occurring after 3 months of treatment. Median time to return to a normal or mild neuropathy score was 3 months. Myelosuppression was the second most common AE occurring in 48% of cases. Although these AE led to frequent treatment interruption (66% of cases had treatment interruption) all 109 participants completed 26 weeks of treatment. In the context of TBM, where morbidity and mortality are high, the risk-benefit of this potent antituberculous agent with good CNS penetration requires further evaluation in the context of a phase II safety trial.

### Aspirin

Aspirin (ASA) is a safe, widely available and inexpensive drug with effects on the pathogenic processes recognised as integral to the pathogenesis of TBM and its complications ^18, 19^. In a placebo-controlled trial of ASA in 118 adult patients with TBM in India, 150mg daily ASA was associated with a significantly lower 3-month mortality and a lower incidence of stroke albeit not significant ^20^. Following this a similar study in South Africa randomized 146 children with TBM to receive low dose (75mg/24hours) (n=47) or high dose (1000mg/24 hours). In this trial there was no significant effect of ASA on mortality however there was a significant reduction on incidence of new hemiplegia in those receiving high dose ASA ^21^. In a recent study in Vietnam, HIV-1 uninfected individuals with TBM received ASA in addition to standard care. Patients were randomised to receive either placebo, ASA 81mg OD or ASA 1000mg OD for 56 days. The pre-specified sub-analysis of results demonstrated a potential reduction in new infarcts and deaths by day 60 in patients with microbiologically confirmed TBM receiving 1000mg OD of ASA ^22^. Its safety for use in HIV-1 infected individuals with TBM, particularly when used in combination with an intensified antituberculous regimen, has yet to be investigated. 

### High dose rifampicin

Rifampicin (RIF), one of the four first line treatments for TBM demonstrates poor penetration of the blood brain barrier (BBB) with total concentrations in CSF only 10–20% of that reached in plasma ^23^. *In vitro*, animal and early bactericidal activity studies suggest that the standard 600mg once daily dose is at the lower end of the dose response curve ^24^. This has prompted a series of studies in both pulmonary and extrapulmonary tuberculosis investigating the safety and efficacy treatment regimens containing higher doses of RIF ^25– 32^. None of these studies have detected a significant safety signal thereby supporting the safety of RIF up to doses of 35mg/kg. Similarly, they provide evidence to suggest superior efficacy when used at a dose of 35 mg/kg compared to 20 mg/kg ^28, 29, 31, 32^.

In TBM, the use of higher RIF doses is appealing given its incomplete penetration into the central nervous system. In 2013 an open-labelled randomized phase 2 study in 60 Indonesian adults with TBM showed a 50% reduction in mortality with higher dose intravenous RIF (13 mg/kg, which equates to an approximate oral dose of 20mg/kg) compared with standard dose oral therapy ^33^. This intensified treatment did not result in increased toxicity and was associated with a substantially lower 6-month mortality. A subsequent large randomised placebo-controlled trial in Vietnam evaluated a combined regimen of oral RIF 15 mg/kg plus levofloxacin. Unlike the previous trial using intravenous therapy (at higher equivalent oral doses of 20 mg/kg) there was no effect of mortality ^25^. These results, plus evidence from pre-clinical studies and pulmonary TB, provide adequate justification to systematically assess the effect on outcomes in TBM with RIF doses > 20 mg/kg.

The proposed trial combines three drugs for which there is sufficient evidence to suggest adequate safety profiles and potential benefit in a condition in which there is high mortality and inadequate treatment. Their safety in combination and in the context of HIV-1 co-infection requires careful evaluation in a controlled Phase II trial before this regimen can be tested in the context of a phase III clinical trial.

Our primary hypothesis is that increased dose RIF plus LZD and ASA can be safely added to standard therapy for HIV-1-associated TBM.

## Methods

This protocol is reported in line with the Standard Protocol Items: Recommendations for Interventional Trials (SPIRIT) guidelines ^34^.

The primary aim of this study is to investigate the safety of enhanced antimicrobial therapy including increased dose RIF and LZD with or without adjunctive ASA added to standard therapy for TBM in HIV-1 infected adults.

The secondary aims are:

a. To determine CSF *M.tb* culture positivity and Gene Xpert® Ultra positivity at baseline and at 3 and 28 days post treatment by allocation.
b. To evaluate the effect of ASA and enhanced TB treatment on the incidence of immune reconstitution inflammatory syndrome (IRIS) in participants starting ART
c. To evaluate the effect of high dose RIF and LZD with and without ASA on CNS imaging (CT, MRI and MR Spectroscopy) in conjunction with clinical, immunological and transcriptional profiling.
d. To determine i) whether host genotype, including leukotriene A4 hydrolase (LTA4H) genotype, influences therapeutic effect of ASA in HIV-TBM and ii) the pharmacogenetic influence on RIF and LZD exposures and toxicity.

Three sub studies will recruit all consenting participants with the following aims:

### Sub study 1 ( *Pharmacokinetic-pharmacodynamic*)

1. To describe the plasma and CSF PK of LZD and high dose RIF.

2. To evaluate the relationship between drug exposures, toxicity and efficacy.

3. To compare exposures between intravenous and oral RIF administration.

4. To investigate the impact of high dose RIF on LZD availability.

### Sub study 2 ( *Pathogenesis*)

1. To evaluate the effect of high dose RIF and LZD, with and without ASA on the transcriptional signature derived from whole blood and CSF RNA sequencing, as well as the metabolomic and proteomic profiles, in TBM.

### Sub study 3 ( *Neurocognitive and functional outcomes*)

1. To describe the frequency and characteristics of neurocognitive impairment following HIV-associated TBM

2. To compare neurocognitive outcomes with: i) presence and location of structural abnormalities on magnetic resonance imaging, ii) radiological makers of metabolic dysfunction on magnetic resonance spectroscopy, iii) *in vivo* markers of neurodegeneration and brain injury within the central nervous system

3. To quantify the functional impairment (including effect on quality of life) of TBM associated neurocognitive impairment

A strategic aim of LASER-TBM is to serve as a planning study to generate data which will inform a planned phase 3 RCT of intensified treatment in TBM (INTENSE-TBM). Data from LASER in particular i) pharmacokinetic data on exposure in intravenous versus high oral dose rifampicin and ii) safety data to exclude any signal which would preclude commencement of INTENSE-TBM will in part dictate the resulting sample size. If no safety signal is detected, and PK endpoints met with adequate power then LASER-TBM recruitment may cease prior to the maximum sample size of 100 participants to allow timely commencement of INTENSE-TBM.

## Study design, recruitment and duration

**Figure 1.**
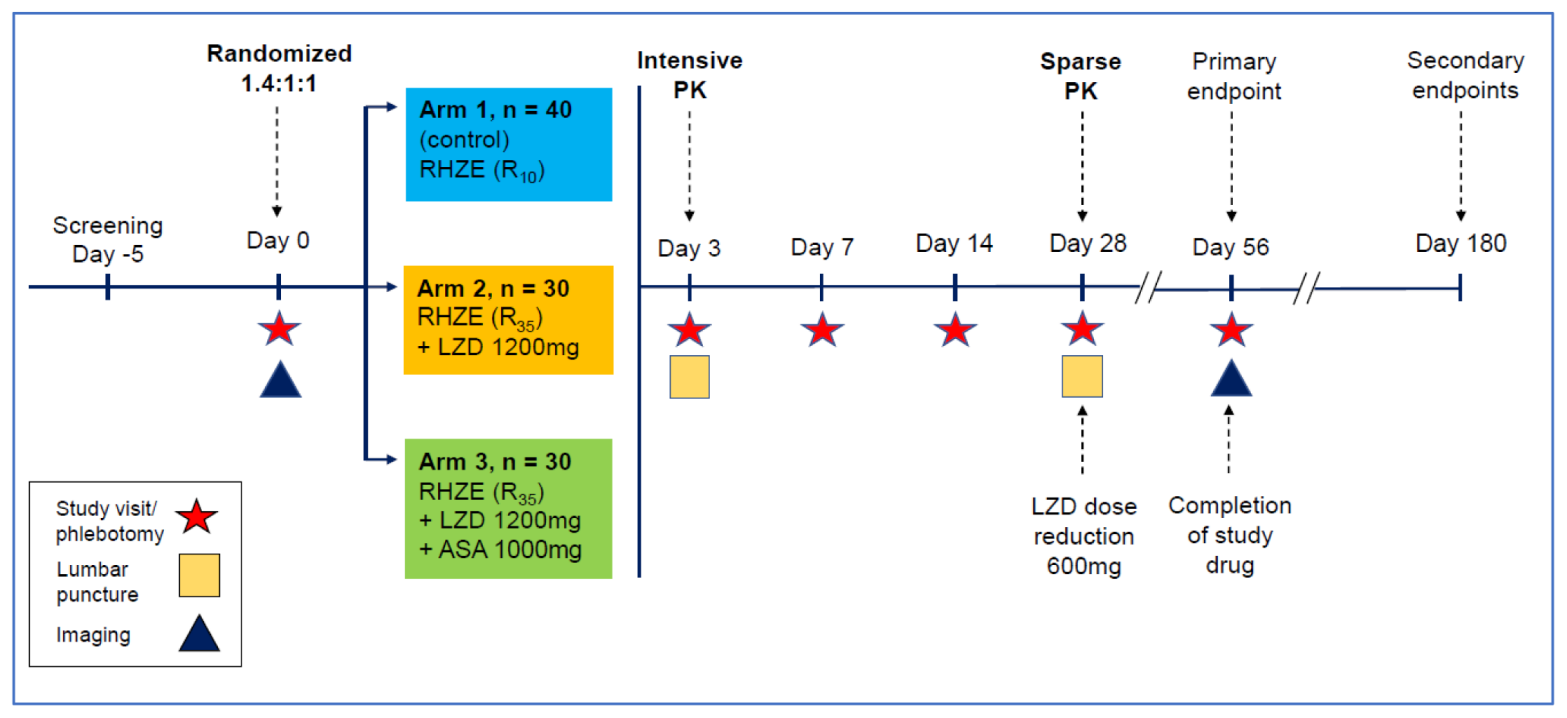
Study design schematic describing randomisation to study arms, treatment intervention per am, visit schedule, overview of clinical procedures and timepoints relating to primary and secondary endpoint data collection. RHZE: Rifampicin, Isoniazid, Pyrazinamide, Ethambutol; R _10_: Rifampicin 10mg/kg/day; R _35_: Rifampicin 35mg/kg/day; LZD: Linezolid; ASA; Aspirin.

LASER-TBM is a parallel group, randomised, multi-arm Phase 2A trial evaluating the safety of increased dose RIF plus LZD, with or without ASA, for the treatment of HIV-infected adults with TBM ( Figure 1). HIV-1 infected adults with newly-diagnosed TBM (up to n = 100) will be recruited from five public-sector hospitals across South Africa. Participants will be randomised in a 1.4:1:1 ratio across two experimental (n = 30 each) and one standard of care (n = 40) arms ( Figure 1). Relatively more participants will be randomised to the control group to account for the higher mortality anticipated in the standard of care arm. 

**Figure 2.**
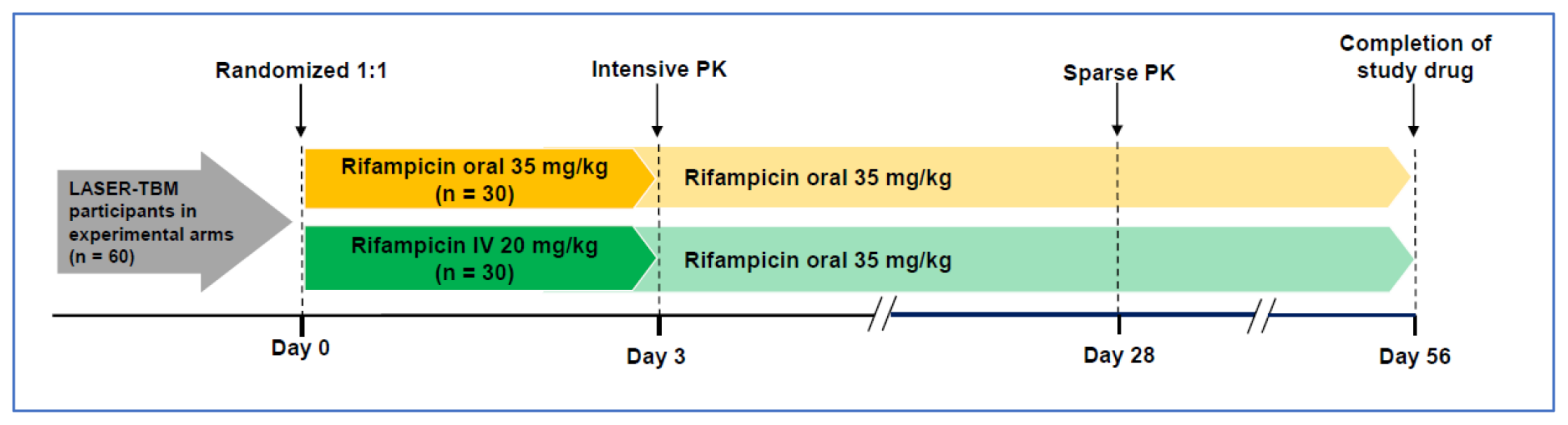
Schematic to describe second randomisation to intravenous rifampicin (IV RIF). All consenting LASER-TBM participants in experimental arms (n = 60) will undergo a second randomisation to receive either oral (35mg/kg) or IV (20mg/kg) RIF, together with linezolid (LZD) (with or without aspirin), at the time of study entry. The second randomisation will take place at the time of study entry, prior to receipt of study drug. Randomisation will be done in a 1:1 ratio using an electronic randomization tool, and fully integrated with main trial procedures). Due to the nature of the intervention, and because the outcome measure is a pharmacokinetic (PK) endpoint, allocation of IV versus oral RIF will be unblinded. Study drug will be stored at site pharmacies and administered as an infusion, in accordance with instructions in the package insert and trial standard operating procedures (SOP), by nursing staff of the trial.

Treatment will be provided in all arms for 56 days, after which participants will be referred back to public sector facilities to complete standard therapy for HIV-associated TBM. All participants will receive antitubercular chemotherapy as well as corticosteroids as per standard practice. Participants allocated to experimental arms 2 and 3 will receive additional RIF (total oral dose 35 mg/kg/day once daily) for 56 days plus oral LZD 1200mg once daily for the first 28 days, reduced to 600 mg daily for the next 28 days. Those randomised to experimental arm 3 will also receive oral aspirin 1000 mg once daily for 56 days. A second randomization will take place before receipt of study drug for participants in the experimental arms (n = 60) to receive either oral (35 mg/kg) or intravenous (20 mg/kg) RIF (see Figure 2). This will be continued for 3 days, after which all participants will receive oral RIF for the remainder of the intervention period (53 days). 

**Figure 3.**
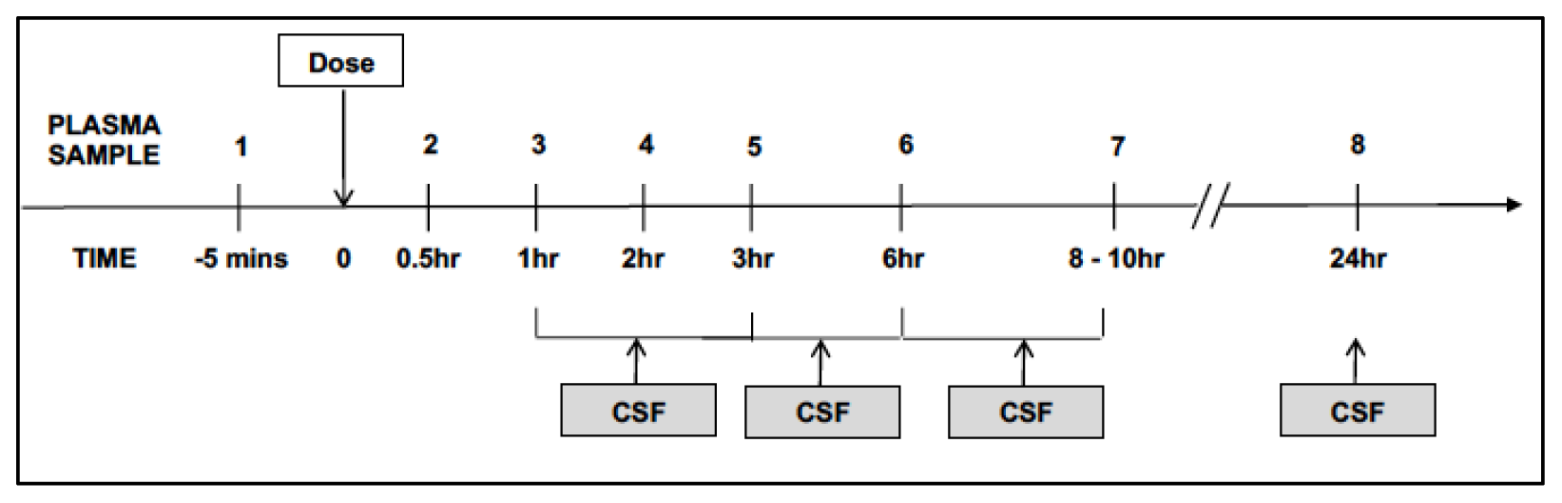
Schematic to summarise intensive pharmacokinetic (PK) sampling schedule. All participants (n=100) will be offered participation in the intensive sampling component of the PK sub-study at the time of randomization to the main study. Intensive plasma sampling will take place at the Day 3 study visit. Serial venous blood samples will be collected through a peripheral intravenous catheter pre-dose, and at 0.5, 1, 2, 3, 6, 8 - 10, and 24 hours after witnessed drug intake and an overnight fast. Sparse sampling will be performed at Day 3 for participants who decline intensive sampling or in whom this fails.

There are six scheduled study visits, which will occur at study sites or affiliated stepdown facilities. Visits will involve clinical history, examination, phlebotomy, lumbar puncture and brain imaging at the timepoints shown in Figure 1. In those who consent, intensive PK sampling will take place at day 3 (see Figure 3). Trial participation will be for 180 days post-randomisation: primary safety endpoints and secondary efficacy endpoints will be evaluated at day 56; additional secondary endpoints will be evaluated at day 180 through record review.

**The primary endpoint of the study is:** The incidence of solicited treatment-related AE (see Table 1) and death at 56 days associated with increased dose RIF plus LZD with or without adjunctive ASA, when administered alongside standard antitubercular therapy.

**Table 1.** Solicited treatment related adverse events, objective measures for assessment and management plan in each setting. Grade relates to Division of AIDS (DAIDS) criteria ^37^. ASA, Asprin; RIF, Rifampicin; LZD, Linezolid.

Adverse Event (responsible study drug)	Objective measure	Parameter	Management
Gastrointestinal haemorrhage (ASA)	Clinical and laboratory measures suggesting GI haemorrhage.	i) Vomiting fresh or changed blood of any volume, ii) Melena, iii) Unexplained drop in Hb concentration of >2g/L or iv) > 5mls of fresh or changed blood aspirated from nasogastric tube.	Discontinue study drug permanently.
Intracerebral haemorrhage (ASA)	Radiological evidence of haemorrhage.	CT or MRI finding	Discontinue study drug permanently.
Transaminitis (RIF)	alanine tranferase (ALT), bilirubin	New Grade 3 or above	Discontinue study drug (and other potentially hepatotoxic agents). Place on alternative treatment for TBM if background regimen affected. Re-test every 2 days. Resume study drug with an escalating dose rechallenge once ALT < 100 IU and total bilirubin within normal range.
Anaemia (LZD)	Hemoglobin (Hb)	New Grade 3	Discontinue study drug (plus any other myelosuppressive drugs as appropriate). Consider transfusion with packed cells or erythropoietin therapy. Monitor Hb twice weekly. Restart at 50% dose once Hb ≥ 8 mg/dL
		New Grade 4	Discontinue study drug permanently. Consider hospital admission and/or transfusion with packed cells or erythropoietin therapy. Re-test every 2 days.
Neutropenia (LZD)	Neutrophils	New Grade 3	Discontinue study drug. Monitor white cell count (WCC) every week. Restart at 50% dose once neutrophil count 0.5 x 109 cells/L.
		New Grade 4	Discontinue study drug permanently. Consider therapy with GM-CSF. Monitor WCC every 1 – 2 days.
Thrombocytopenia (LZD)	Platelet (Plt) count	New Grade 3	Discontinue study drug (plus any other myelosuppressive drugs as appropriate). Monitor Plt count twice weekly. Restart at 50% dose once Plt count > 50 x109 cells/L.
		New Grade 4	Discontinue study drug permanently. Consider hospital admission and/or transfusion pooled Plts. Re-test every 1 - 2 days.
Peripheral Neuropathy (LZD)	Full neurological history and examination, Brief Peripheral Neuropathy Score (BPNS) and Modified Total Neuropathy Score (mTNS)	Change in clinical history of examination resulting in: I) 1 grade increase in BPNS ii) 2 grade change in any modality on mTNS	Review with a view to discontinuing study drug (plus any other neuropathic drugs like INH). Consider restarting at 50% dose once completely resolved.
Optic Neuropathy (LZD)	14-plate Ishihara Test, visual acuity measured by logMAR chart	Change in score of 2 on 14-plate Ishihara Colour Test or new or worse logMAR score of 0.2	Stop study drug and EMB and refer for formal ophthalmological assessment. If assessment consistent with optic neuritis do not restart drug.

**Secondary study endpoints are:**

• Death and severe disability (Modified Rankin Scale Grade 5) at 56 days ( Box 1).

**Table:** Box 1. Modified Rankin score

**SCORE**	**DESCRIPTION**
0	No symptoms
1	No significant disability. Able to carry out usual activities, despite some symptoms.
2	Slight disability. Able to look after own affairs without assistance, but unable to carry out all previous activities.
3	Moderate disability. Requires some help, but able to walk unassisted.
4	Moderately severe disability. Unable to attend to own bodily needs without assistance, and unable to walk unassisted.
5	Severe disability. Requires constant nursing care and attention, bedridden, incontinent.
6	Dead

• Death at 56 and 180 days.

• Disability at 56 and 180 days, stratified by baseline MRC grade.

• Grade 3 or 4 AE.

• Permanent discontinuation of experimental drugs.

• Severity and frequency of haematologic and neurologic AE related to LZD use.

• Severity and frequency of major bleeding (gastrointestinal and intracerebral) related to ASA use.

• *M. tb* culture status and time to positivity in automated liquid culture (MGIT) and Gene Xpert® Ultra cycle threshold (C _t_) values at days 28 and 56.

• The occurrence of TBM-IRIS assessed by the modified International Network for the Study of HIV-associated IRIS (INSHI) criteria ^35^.

• MRI and CT changes at day 56.

Study participants for LASER TBM must be adults (aged 18 years or over), with proven HIV-1 seropositivity, and a diagnosis of TBM meeting criteria for ‘possible’, ‘probable’ or ‘definite’ as per the published consensus definition ^36^.

Potential participants will be excluded if they meet any of the exclusion criteria outlined in Box 2.

**Box 2. Eligibility criteria**

**Inclusion criteria**

•   Age ≥18 years

•   proven HIV-1 seropositivity

•   Diagnosis of ‘possible’, ‘probable’ or ‘definite’ TBM

**Exclusion criteria**

•   Rifampicin-resistant *M.tb* detected on any clinical specimen;

•   History of allergy or hypersensitivity to RIF, isoniazid, ethambutol, pyrazinamide, LZD or ASA;

•   Received more than 5 days of antitubercular therapy in the 30 days prior to screening;

•   Receipt of regular daily ASA or NSAID prior to TBM diagnosis

•   CSF unobtainable by lumbar puncture or another procedure;

•   Evidence of bacterial or cryptococcal meningitis;

•   Severe concurrent uncontrolled opportunistic infection including, but not limited to, active cytomegalovirus-associated disease, Kaposi sarcoma, *Pneumocystis jirovecii* pneumonia, HIV related or unrelated malignancy, or gastrointestinal bleeding;

•   Any other form of immunosuppressive therapy, including antineoplastic and biologic agents, apart from corticosteroids;

•   More than 17 weeks pregnant at baseline;

•   Peripheral neuropathy scoring Grade 3 or above on the BPNS;

•   Any disease or condition in which the use of the standard anti-TB drugs (or any of their components) are contraindicated. This includes, but is not limited to, allergy to any TB drug or their components;

•   The presence of one or more of the following:

-   Estimated glomerular filtration rate (eGFR) < 20ml/min/1.73 m2 *

-   INR > 1.4 and/or clinical evidence of liver failure or decompensated cirrhosis;

-   Haemoglobin < 8.0 g/dL;

-   Platelets < 50 x109 /L;

-   Neutrophils < 0.5 x 109 cells/L;

•   Any disease or condition in which any of the medicinal products listed in the section pertaining to prohibited medication (See Box 1) is used and cannot be safely stopped;

•   Known or suspected history of drug abuse or any other reason that is, in the opinion of investigators, sufficient to compromise the safety or cooperation of the participant.

*Calculated using the Cockcroft-Gault equation; INR: International normalised ration; BPNS: Brief peripheral neuropathy score; NSAID: Non-steroidal anti-inflammatory drug.

## Recruitment, randomisation, retention and withdrawal

Recruitment will be from inpatients at the participating hospital sites in South Africa (Groote Schuur Hospital, Mitchells Plain Hospital, New Somerset Hospital and Livingstone Hospital). Suitable patients will be identified by attending ward doctors and co-investigators at each site and referred to the study staff for screening.

Participant identification numbers (PID), assigned at the screening visit, will be used throughout the study. After signing the informed consent document, eligible participants will be randomised to one of the treatment arms using a pre-generated electronic randomisation list created within Microsoft Excel prior to commencement of the study. The randomisation list will be generated and updated by the trial pharmacist who will have no direct contact with trial participants or involvement with the assessment for eligibility in the trial. The second randomisation to IV or oral RIF will take place immediately, prior to receipt of study drug, for all participants allocated to experimental arms. The trial is open-label, and regimens will not be masked therefore all study team members involved in the participants care will be aware of the study arm allocation.

All trial procedures will take place in hospital during the admission period. The decision to discharge trial participants will be made by clinical, and not trial, staff. Site-specific standard operation procedures (SOP) will be developed for trial follow up visits following discharge or referral to a stepdown facility. 

A participant will be withdrawn from the study if:

• The initial *M.tb* strain is found to be RIF-resistant on confirmatory testing;

• HIV-1 result is found to be negative on confirmatory testing;

• An alternative diagnosis is established within 5 days of antitubercular treatment initiation which leads the treating physician to discontinue antitubercular therapy;

• Withdrawal of informed consent.

Participants who withdraw consent prior to completion of the study will not undergo any further study procedures or data collection. In such cases, consent for the use of data collected prior to withdrawal of consent will be sought from the withdrawing participants. There will be no replacement for participants withdrawn from the trial.

## Interventions

### Study drug regimens

Participants enrolled to the study will receive study drug regimens as outlined in Table 2. Dosing of the RHZE fixed dose combination (FDC) will be according to World Health Organisation (WHO) weight bands ^38^. Study drugs will be given orally, either as tablets/capsules or crushed, depending on the clinical circumstances. Half of participants in experimental arms will be randomised to receive IV RIF for the first 3 days of therapy and switched to the oral formulation thereafter. Study drugs will be prescribed by trial doctors, packaged and distributed by trial pharmacists.

**Table 2.** Details and dosing of study drug regimen - provided for 56 days post randomisation.

	Drug					
Arm	RIF	INH	EMB	PZA	LZD	ASA
**1**	10 mg/kg O.D.	5 mg/kg O.D.	15 mg/kg O.D.	25 mg/kg O.D.		
**2**	35 mg/kg O.D.	5 mg/kg O.D.	15 mg/kg O.D.	25 mg/kg O.D.	1200 mg O.D. (28 days) then 600 mg O.D. (28 days)	
**3**	35 mg/kg O.D.	5 mg/kg O.D.	15 mg/kg O.D.	25 mg/kg O.D.	1200 mg O.D. (28 days) then 600 mg O.D. (28 days)	1000 mg O.D.

O.D.: Once daily.

### Oral RIF dosing bands

Weight based dosing to achieve equitable exposure across weight bands was based on simulations as described previously ^39^. These are described in Table 3A.

**Table 3A.** Weight bands for oral rifampicin (RIF) dosing.

LASER-TBM bands	Band 1	Band 2	Band 3	Band 4	Band 5
**Weight range**	30 – 37 kg	38 – 54 kg	55– 65 kg	66 – 70	> 70 kg
**R _10_HZE (WHO)**	300	450	600	600	750
**R _25_ additional**	1200	1350	1500	1650	1950
**Total RIF (~35 mg/kg)**	1500	1800	2100	2250	2700

### Intravenous RIF

Participants allocated to experimental arms will be randomised (1:1) to receive either oral RIF 35 mg/kg or IV RIF 20 mg/kg once daily for the first 3 days of therapy (in addition to HZE and LZD with or without ASA, according to the experimental arm). Those randomised to IV RIF will receive the full RIF dose intravenously, plus additional antitubercular drugs as individual ablets (at standard doses). IV RIF will be administered as an infusion as per the package insert and according to a detailed SOP. These are described in Table 3B.

**Table 3B.** Weight bands for intravenous rifampicin (RIF) dosing.

	Band 1	Band 2	Band 3	Band 4	Band 5	Band 6
Weight range	30 – 33 kg	34 – 37 kg	38 – 54 kg	55– 65 kg	66 – 70 kg	> 70 kg
HZE tabs	2	2	3	4	4	5
R _20_ IV	900	1050	1200	1350	1500	1650
Total RIF	900	1050	1200	1350	1500	1650

## Concomitant medications

### Corticosteroids

All participants will receive corticosteroids for the first 8 weeks of TBM treatment as used in a randomised controlled trial demonstrating mortality benefit in patients TBM ^5^.

### Antiretroviral therapy (ART)

ART will be commenced by treating clinicians after 4–6 weeks of antitubercular therapy in all participants based on the single randomised controlled trial of ART timing in TBM, which showed no benefit for earlier ART ^40^. If available, a dolutegravir-based regimen will be used in accordance with international ^41^ and local guidelines ^42^.

### Gastric protection

Participants can be prescribed omeprazole 40mg daily. A higher starting dose of 40mg OD was selected to account for the interaction between proton pump inhibitors (PPI) with rifampicin via CYP2C19 and CYP3A4 leading to reduction in levels of the PPI. In participants with persistent symptoms the dose will be titrated to 80mg daily and gastroscopy considered. Although the study initially planned to use ranitidine for this indication, this was withdrawn as a concomitant medication due to concerns over a potential contamination with the probable carcinogen N-nitrosodimethylamine (NDMA) ^43^, making the medication unavailable for use in South Africa. 

### Pyridoxine

Participants will receive pyridoxine supplementation as per the South African guidelines for prevention of anti-tuberculosis drug-related peripheral neuropathy ^44^.

### Disallowed medications

The medicines listed in Box 3 have been shown to interact with study drugs and are therefore contraindicated for concomitant use during the study.

**Box 3. Contraindicated medications for study participants**

**Tricyclic antidepressants:** Amitriptyline, Amoxapine, Clomipramine, Desipramine, Doxepin, Imipramine, Nortriptyline, Protriptyline, Trimipramine. **Selective Serotonin Re-uptake Inhibitors (SSRI’s):** Citalopram, Escitalopram, Fluoxetine, Fluvoxamine, Paroxetine, Sertraline. Serotonin-Noradrenaline Re-uptake Inhibitors (SNRI’s) Venlafaxine, Duloxetine, Levomilnacipran, Milnacipran. **Serotonin Receptor Agonist**, Buspirone, **Mono-amine Oxidase Inhibitors (MOAIs**), Isocarboxazid, Nialamide, Phenelzine, Tranylcypromine, Selegiline, Rasagaline, Toloxatone. **Reversable MOA Inhibitors (RIMAs):**Moclobemide, Pirlindole. **Migraine medications**: Triptans. **Macrolide antibiotics**: Clarithromycin, Erythromycin, Troleandomycin. **Opiate analgesics**: Methadone, Tramadol, Pentazocine. **Stimulants**: MDMA (ecstacy), Cocaine, Methamphetamine **Hormonal treatment**: Gestodene, Testosterone. **Other medications**: Antiretrovirals – Atazanavir, Anti-arrhytmic – Quinidine, Anti-malarial – Quinine, Chemotherapy – Doxorubicin, Asthma –Furafylline, Hypertension – Hydracarbazine, Antifungal – Ketokonazole, Amino-acid - Tyramine

**Bold** represents class of drug. Underlined medications represent commonly used medications in South Africa.

## Study procedures, schedule and clinical assessments

Participants will undergo six scheduled study visits after screening, plus ascertainment of vital status and disability assessment at 6 months. Table 4 describes planned investigations at each study visit.

**Table 4.** Planned study assessments and procedure per study date.

Visit (window in days)	SCR	ENR	Day 3 (+/- 1)	Day 7 (+/- 2)	Day 14 (+/- 2)	Day 28 (+/- 3)	Day 56 (+/- 4)	Day 180
**Bedside**								
Study Informed Consent	x	x						
Vital Signs	x	x	x	x	x	x	x	
Medical History	x	x	x	x	x	x	x	x
Physical Examination	x	x	x	x	x	x	x	
BPNS and mTNS	x							
Modified Rankin Score		x		x	x	x	x	x
Insomnia Questionnaire						x	x	
MOCA, IHDS, EQ5d5L							x	
Neurocognitive mini-battery								x
AE/SAE, Adherence Monitoring		x	x	x	x	x	x	
Randomisation and treatment assignment		x						
**Blood**								
Weight		x	x	x	x	x	x	
Haematology: FBC and white cell differential								
Biochemistry: Creatinine, eGFR, electrolytes, LFTs	x		x	x	x	x	x	
INR	x		x			x		
HIV-1 ELISA +/- HIV Rapid Test (x2) if required	x							
CD4+ count, HIV Viral Load		x						
Plasma for PK sub-study (sparse sampling)						x	x	
Plasma for PK sub-study (intensive sampling – if consented)			x					
Stored plasma for immunological, proteomic and metabolomic profiling		x	x	x	x	x	x	
PBMC for storage		x	x			x		
Whole blood for RNA extraction		x	x	x	x	x	X	
Whole blood for DNA extraction (if consented)		x						
**Urine**								
Urine for pregnancy test	x							
Urine for storage		x			x		x	
**Cerebrospinal Fluid Analysis**								
Cell count, MC+S, TB culture, GeneXpert Ultra (inc Rif resistance)			x			x		
Biochemistry: protein and glucose			x			x		
Stored CSF for immunological, cellular, proteomic and metabolomic profiling;			x			x		
CSF for RNA extraction								
CSF for PK sub-study			x			x		
**Imaging**								
MRI Head, or CT Head if MRI not tolerated (+/- 5 days)		x					x	

SCR: Screening; ENR: Enrolment; AE: Adverse Event; BPNS: Brief Peripheral Neuropathy Score; CSF: Cerebrospinal Fluid; CT: Computerised Tomography; FBC: Full Blood Count; LFT: Liver Function Tests; IHDS: International HIV Dementia Score; MC+S: microscopy, culture and sensitivity; MOCA: Montreal Cognitive Assessment; mTNS: modified Total Neuropathy Score; MRI: Magnetic Resonance Imaging; PAOFI: Patients Assessment of Own Functioning Inventory; PBMC: Peripheral Blood Mononuclear Cells; PK: Pharmacokinetic;.

### Clinical assessment

Clinical assessment will include clinical history, conscious level by GCS, presence of new or ongoing focal neurological deficit, all adverse events, new medications started and adherence to drugs. The neurological examination at D180 is extended to assess for such as language, visuospatial deficit, visual agnosia and praxis: focal neurocognitive deficits which may be present in people with TBM.

### Modified Brief Peripheral Neuropathy Score

This purely clinical early screening tool was adapted from the subjective peripheral neuropathy score (SPNS) validated for the assessment of HIV associated distal sensory polyneuropathy (DSP) ^45^ and used previously in trials to assess LZD toxicity.

### Modified Total Neuropathy Score

This screening tool, initially developed for the assessment of chemotherapy induced neuropathy ^46^, has been modified for use in the HIV research setting where it has shown acceptable sensitivity and specificity (85% and 80% respectively) ^47^. Prior studies have used a simplified 16-point ^48^ or 20-point clinical scoring system ^49^ as markers of HIV-associated DSP severity.

### Insomnia Severity Index

All participants will complete the Insomnia Severity Index (ISI) at the Day 28 and 56 visits. The ISI is a brief screening measure of insomnia which has been validated for use in insomnia research ^50^. Dolutegravir has been associated with neurotoxicity presenting with neuropsychiatric symptoms such as insomnia ^51^, and this will be assessed as part of the PK/PD assessment.

### Measures of neurocognitive function

These measures where possible will be done in the participants preferred language.

*i) Montreal Cognitive Assessment*
  The Montreal Cognitive Assessment (MoCA) assesses six broad domains of ability and neurocognitive function ^52^ and has been used to screen for cognitive impairment in previous studies within South Africa ^53^. This will be carried out at day 56 and day 180.
*ii) Cognitive Assessment Tool-Rapid Version (CAT-Rapid).* This instrument includes four questions about subjective cognitive complaints, as well as tasks assessing learning and memory and cognitive sequencing. CAT-rapid was developed in South Africa (Joska *et al*., 2016), in response to the need to develop a brief screening tool that includes functional symptom questions and a measure of executive function. The CAT-rapid incorporates aspects of the International HIV Dementia Scale and includes four symptom questions, as well as tasks assessing learning and memory and cognitive sequencing.
*iii) Brief neuropsychological battery*
  Neuropsychological testing will be carried out by a trained neuropsychometric tester and clinical research worker at day 180 and will include a neurocognitive test battery and assessment of contributing mental health symptoms. The neurocognitive battery comprises 12 standardised tests, each of which assesses performance in one of six cognitive domains commonly affected by TBM. The domains, tests, and outcome variables are: (1) Executive Functioning: Color Trails Test 2 (CTT2) - completion time; Wisconsin Card Sorting Test (WCST) - perseverative errors; (2) Learning and Memory: Hopkins Verbal Learning Test-Revised (HVLT-R) - total learning total and delayed recall total; Brief Visuospatial Memory Test-Revised (BVMT-R) - total learning total and delayed recall total; (3) Generativity/fluency: category fluency - total number of animals / total number of fruits and vegetables named in 1 minute; (4) Attention/Working Memory: Wechsler Adult Intelligence Scale-Third Edition (WAIS-III) Digit Span - total score; (5) Processing Speed: CTT1 - completion time; Wechsler Adult Intelligence Scale-Third Edition (WAIS-III) Digit Symbol Coding - total score; WAIS-III Symbol Search- total score; (6) Motor Function: Grooved Pegboard Test (GPT) dominant (DH) and nondominant hand (NDH) - completion time; Finger Tapping Test (DH and NDH) - completion time. Tests were administered in either English or Xhosa depending on the participant’s preference. Mental Health measures are the Centre for Epidemiological Studies-Depression (CES-D), State Trait Anxiety Inventory-trait (STAI-trait), Alcohol Use Disorders Identification Test (AUDIT) and Drug Use Disorders Identification Test (DUDIT).

### Measures of functional status

*i) Modified Rankin Score*
  The MRS, a commonly used clinical outcome measure for patients suffering from stroke ^54^, has demonstrated good inter-rater reliability ^55^ and is the most commonly used outcome measure to assess neurological disability in TBM ^56^.
*ii) Modified Patients Assessment of Own Functioning Inventory (PAOFI)*
  This patient reported outcome measure is designed to evaluate a patient’s sense of his or her functional capacity in everyday activities concerning memory, language and communication, use of hands, sensor perception, higher level cognitive and intellectual functions, and work/recreation ^57^.
*iii) Lawton Instrumental Activities of Daily Living-South Africa*
  This test is designed to assess independent living skills, considered more complex than basic activities of daily living. This was developed in 1969 ^58^, but since modified for use in the South African context ^59^.

### Blood

Testing will be done at specified timepoints (as per Table 4), and may be repeated to follow-up on abnormal results, for example after occurrence of an AE. Samples for haematology and biochemistry and HIV testing will be processed in National Health Laboratory Service (NHLS) laboratory according to local SOPs. Samples for non-clinical assays (immune markers, RNA, metabolomics, proteomics) and future use will be collected and transported to the Institute of Infectious Disease and Molecular Medicine at the University of Cape Town (UCT) for processing and storage. PK samples will be centrifuged *within an hour* of being taken at 1500 x g at room temperature for 10 minutes, the plasma aliquoted into cryovial tubes, stored at -80ᵒC and transported to UCT Clinical Pharmacology laboratory for storage and processing.

### Urine

Bedside pregnancy testing will be done on urine at screening. Urine will be sent to chemistry laboratories for osmolality and electrolyte testing in the context of hyponatraemia at the discretion of the investigator. Further urine will be collected for biobank storage.

### Cerebrospinal fluid analysis

CSF will be obtained via lumbar puncture at Days 3 and 28, in accordance with a detailed SOP. CSF collected for diagnosis in routine care (baseline) will be retrieved where possible. Routine microbiology, cell count, and biochemistry will be done in NHLS laboratory according to local SOPs. Samples for non-clinical testing (RNA sequencing, metabolomic and proteomic analysis and immunological assays) will be collected and transported to the IDM at UCT for processing and storage. CSF to determine drug concentrations for the PK study will be frozen at -80 degrees immediately following collection and transported to UCT Clinical Pharmacology laboratory for storage and processing.

### Magnetic resonance imaging (MRI)

MRI scans will be performed in all participants who can tolerate or access the investigation at baseline and day 56. A 3-Tesla (3T) MRI scanner located at Groote Schuur Hospital will be used for all imaging in the Cape Town area, whilst a 1.5T scanner located at Livingstone Hospital will be used for participants recruited in the Port Elizabeth Area. Gadolinium enhanced imaging will be performed on participants with eGFR < 30mL/min/1.73m ^2^.

Image sequences will include the following:

• T1 weighted sequences with or without gadolinium

• T2 weighted sequences

• Diffusion weighted images (DWI)

• Susceptibility weighted images (SWI)

• T2 Fluid-attenuated inversion recovery (FLAIR)

• Point resolved spectroscopy (PRESS/MEGA-PRESS) to estimate brain metabolite changes

### Computed tomography (CT)

If participants are unable to tolerate or access MRI, CT will be used as an alternative imaging method at the same time points as stated for MRI. Participants with eGFR > 30 mL/min/1.73m ^2^ will have contrast enhanced imaging. Pre- and post- contrast sequences will be available for analysis. A standardised reporting form including positive and negative radiological findings will be used. 

## Statistical considerations

### Sample size

The total number of participants required for primary safety analysis is 100. This encompasses:

Arm 1 - control - (standard-of-care): 40

Arm 2 - experimental - (standard-of care + high dose rifampicin + linezolid): 30

Arm 3 - experimental - (standard-of care + high dose rifampicin + linezolid + aspirin): 30

### Sample size justification

This phase IIA trial will focus on evaluating adverse events in the experimental arm relative to the standard of care arm. Solicited treatment related AE ( Table 1) plus deaths will be evaluated, and the Data Safety Monitoring Board (DSMB) will provide recommendations accordingly. The DSMB will review all safety events and approve the ongoing conduct of the trial. Analyses that will aid their decision-making will be based on several sources: 

First, a test of proportions will compare the AE rates between the standard-of-care arm and the experimental arms. Concerns about a worse safety profile will be flagged using a two-sided type I error rate of 0.1. Consider a scenario in which there are 10 out of 20 AE in the standard-of-care arm and 14 out of 20 in the experimental arms. This corresponds to a two- sided p-value of 0.053 using Boschloo’s test and would be reason for the DSMB to consider stopping the trial. 

Similarly, a Bayesian posterior probability (with an uninformative prior) of the probability that the AE rate in the experimental arm is worse than that in the control arm. This will provide an additional means of interpreting the relative results. If this probability is high, the DSMB may recommend stopping the study. For example, in the 10/20 and 14/20 scenario the posterior probability that the experimental arm has a worse rate of AEs is 94%. If the split was 10/20 versus 14/20, this probability would be 89%. The DSMB will be unblinded to safety data after every 15 patients recruited (5 per arm). At each point, absence of a significant safety signal (as outlined in the DSMB charter) will permit ongoing recruitment.

Although there is no power calculation to justify the primary outcome due to the complexity of multiple adverse events of special interests, it was felt that recruitment of up to 100 participants will allow sufficient numbers to permit calculations as detailed above and in doing so reveal a signal in terms of the safety of use of the three investigational products. Further detail is provided in the statistical analysis plan included as extended data ^34^.

### Statistical analysis plan

***Primary analysis of primary endpoint.*** The primary analysis will be performed in the modified intent-to-treat population (those who receive any dose of the study drug). An accompanying sensitivity analysis will be performed in the per-protocol population (those who completed treatment as specified in the protocol). The primary endpoint, occurrence of solicited treatment related adverse events (AESI) or death will be summarised as x/n (%) (number of individuals experiencing any AESI/number of individuals in each group) for each arm. Two sided 95% Wilson confidence intervals for risk differences comparing each of the two investigational arms to the control arm will be reported for descriptive purposes. Fisher’s exact test will be used to make the same comparisons and accompanying two-sided p-values and 95% confidence intervals for the odds ratio will be reported.

***Secondary analyses of primary endpoint.*** A time to event analysis will be performed on time to worst grade (in each individual) AESI or death. Kaplan-Meier curves will summarize the time to worst grade by arm, and comparisons between each investigational arm against the control arm will be made using the log-rank test.

Further statistical analysis plans are detailed in the statistical analysis plan for LASER-TBM co-authored and authorised by the trial statistician (JL) (see data repository) including the planned analysis for the primary, secondary endpoints and and PK endpoints. All analysis will be performed in R (version 4.04) and GraphPad Prism (version 9 for macOS)) software.

## Adverse events

### Assessment of AE

Study participants will be monitored and assessed for new AE at all scheduled study visits (as shown in Table 4). At each visit trial staff will also assess the evolution and outcome of previously recorded AE. Safety monitoring of the study will be performed by a DSMB as described below.

### Severity of AE

All AE will be assessed for severity by study clinical investigators and graded as per the Division of AIDS (DAIDS) criteria ^60^. Each AE will be assigned a grade 1 to 5. For events not included in the protocol-defined grading systems, the following general definitions from grades 1 to 4 will be applied to classify event severity:

Changes in the severity of an AE should be documented to allow an assessment of the duration of the event at each level of intensity to be performed. AE characterised as intermittent require documentation of onset and duration of each episode.

### Solicited treatment related AE

Table 1 lists AE of special interest which are considered ‘solicited treatment related AE’ and therefore comprise primary safety endpoints of this study. These AE are reported regardless of causal relationship to study drugs. For each AE there is a specific objective measure incorporating the DAIDS grading criteria and other parameters of clinical significance. The management of each AE is summarised.

### Management of adverse events

Treatment must be discontinued for safety reasons if any clinical AE, laboratory abnormality, intercurrent illness, or other medical condition or situation occurs such that continued exposure to treatment would not be in the best interest of the participant. Management of solicited treatment related AE is summarised in Table 1; detailed guidance for management of AE is provided in the manual of operating procedures.

## Safety monitoring

### Safety oversight

Safety oversight will be under the direction of an independent DSMB. Comprised of independent internationally-recognised HIV-TB researchers and an independent statistician, the DSMB will meet after each 15 participants enrolled. The DSMB may also decide to convene an unscheduled review if warranted by safety or data quality concerns. The data for review will be prepared by an independent statistician.

The task of the DSMB will thus be to review study recruitment, data quality and trial drug safety and advise the sponsor of major safety issues and data quality issues. The DSMB may advise that trial enrolment should be paused or stopped entirely based on the decisions regarding the frequency and severity of solicited treatment related AE as outlined in Table 1 (‘Solicited Treatment Related Adverse Events’).

### Pausing and stopping rules

Halting rules for safety reasons will be detailed in the DSMB charter. In the event of serious safety concerns, the DSMB chair will consult the full DSMB by email or teleconference. Pending the DSMB response, the chair may use his/her discretion to recommend one or more if the following: Halt in study (arm) enrolment; halt in study (arm) dosing; provision of additional intervention; no action. After review, the DSMB will issue a recommendation to the trial steering committee to continue, modify (one or more arms) or terminate the trial.

## Data access and handling

### Source documents

Source data are original records of clinical findings, observations, or other activities necessary for the evaluation of the trial. Examples of these original documents and data records include, but are not limited to: hospital records, laboratory reports, and radiological images. Case report forms (CRF) may also be acceptable source documents. A complete list of source documents will be created prior to trial initiation.

The following individuals and groups will have access to study records:

- Members of the study team

- Relevant institutional review board (IRB)

- Regulatory agencies (South African Health Products Regulatory Authority - SAHPRA)

- Study Monitor

All site staff, the sponsor, and any sponsor representatives will preserve the confidentiality of all participants taking part in the study in accordance with ICH GCP, applicable South African national and local regulations and (to the extent applicable) the U.S. Health Insurance Portability and Accountability Act of 1996 (HIPAA). Subject to the requirement for source data verification by the study personnel by reference to the participant’s notes confidentiality of all participant identities will be maintained. Only participant study number and initials will be used on the CRF and in all study correspondence, as permitted. No material bearing a participant’s name will be kept on file by the sponsor. The written informed consent will contain a clause granting permission for review of the participants’ source data.

### Data collection, management and storage

Procedures to ensure data quality will be detailed in a data management plan. Data will be collected and captured onto hardcopy CRF on site and then entered into an electronic database. Clinical data will be entered onto paper CRF directly from the source documents on site. CRF will be cross-checked for accuracy, authenticity and completeness at the site by study staff; checks for consistency will be implemented at the data entry level on site and centrally after data entry.

The data will be managed and stored using a GCP-compliant web-based REDCap® database custom-designed for the study. The REDCap® data entry and user permission structures provide auditing trails in line with international requirements. Access to the database is password controlled and will be limited to delegated trial staff with data entry and data management responsibilities.

### Publication of research findings

The definition of publication for this purpose is any public presentation of the data emerging from this study. All unpublished information given to the investigator by the sponsor shall not be published or disclosed to a third party other than to the responsible IRB, within the understanding of the confidentiality of their nature, without the prior written consent of the Sponsor. Results of this research will be submitted for publication as soon as feasible upon completion of the study in the form of a joint publication(s) between the sponsor and investigator(s), including site clinical and laboratory investigators, as appropriate

## Trial committees

A trial management group (TMG) responsible for the day-to-day management of the trial at the UCT CRC includes; National Trial Coordinator (Ms Mpumi Maxebengula), Lead Clinician (Dr Angharad Davis), Research Medical Officers (Dr Cari Stek, Dr Remy Daroowala, Dr Marise Bremer, Dr Stephani Botha, Dr Saalika Aziz), Project Manager (Ms Rene Goliath), Pharmacists (Ms Sonya Koekemoer, Mr Yakub Kadernani). The group will communicate weekly to discuss trial progress.

The trial steering commitee (TSC) is composed of Professor Guy Thwaites (chair, Infectious Disease Physician, University of Oxford), Professor Graeme Meintjes (site principal investigator), Dr Sean Wasserman (site principal investigator), Dr John Black (site principal investigator), Professor Robert J Wilkinson (National Principal Investigator) and Dr Angharad Davis (lead investigator). The role of the TSC is to provide overall supervision for the trial and provide advice through its independent chair. The ultimate decision for the continuation of the trial lies with the TSC.

The Data Safety and Managament Board (DSMB) is composed of Professor David Lalloo (chair, Director of the Liverpool School for Tropical Medicine and a Professor of Tropical Medicine), Dr David Meya (Infectious Diseases clinician, Senior Lecturer at the College of Health Sciences at Makerere University and Adjunct Associate Professor in the Division of Infectious Diseases and International Medicine at the University of Minnesota), Dr Evelyne Kestelyn (Head of the Clinical Trials Unit at the Centre for Tropical Medicine and Global Health, University of Oxford), Dr Maryline Bonnet (Medical Epidemiologist Institute of Research for Development and Epicentre), Dr Angela Crook, (Trial Statistician). The role of the DSMB is to protect and serve LASER-TBM trial patients and to assist and advise the Principal Investigators, so as to protect the validity and credibility of the trial.

## Ethics

The trial has ethics approval from the University of Cape Town Human Research Ethics Committee (293/2018), Walter Sisulu University Human Research Committee (Ref 012/2019) and the South African Health Products Regulatory Authority (reference number 20180622). The trial is registered on the South African National Clinical Trials Register (DOH-27-0319-6230) and Pan African National Clinical Trials Register (PACTR201902921101705).

## Trial sponsor

University of Cape Town (Clinical Research Centre)

Delva Shamley

L51 Old Main Building

Groote Schuur Hospital

Observatory, Cape Town

Tel: 021 650 1975

Email: delva.shamley@uct.ac.za

## Study recruitment sites

### Cape town

Groote Schuur Hospital, Main Road, Observatory, Cape Town, 7925, Republic of South Africa

Mitchells Plain Hospital, 8 A Z Berman Drive, Lentegeur, Cape Town, 7786, Republic of South Africa

New Somerset Hospital, Bay Court, Portswood Road, Green Point, Cape Town, 8001, Republic of South Africa

### Port Elizabeth

Livingstone Hospital, Standford Road, Korsten, Port Elizabeth, 6020, Republic of South Africa

## Version control

Submitted version of the protocol: V6 (dated 11 May 2020).

## Protocol amendment policy

Any change to the protocol will be affected by means of a protocol amendment. The PI, HREC, and sponsor must agree on all amendments. No amendment will be implemented until approved by the relevant authorities and signed by all required parties. Exceptions to this are when the PI considers that the participant’s safety is compromised. No deviations from or changes to the protocol should be initiated without prior written approval from the IRB and regulatory authority. The PI, or designated site staff, is responsible for documenting and explaining any deviations from the protocol. Protocol deviations must be sent to the Sponsor and IRB in accordance with standard procedures.

## Study status

To date the study has enrolled 52 participants.

## Data availability

### Underlying data

No data are associated with this article.

### Extended data

Figshare: SPIRIT Checklist and Statistical Analysis Plan. https://doi.org/10.6084/m9.figshare.14508561 ^34^.

### Reporting guidelines

Figshare: SPIRIT checklist for ‘Study protocol for: Aa phase 2A trial of the safety and tolerability of increased dose rifampicin and adjunctive linezolid, with or without aspirin, for HIV-associated tuberculous meningitis [LASER-TBM]’. https://doi.org/10.6084/m9.figshare.14508561 ^34^.

Data are available under the terms of the Creative Commons Attribution 4.0 International license (CC-BY 4.0).
