# Supplementary material for: Study protocol for a phase 2A trial of the safety and tolerability of increased dose rifampicin and adjunctive linezolid, with or without aspirin, for HIV-associated tuberculous meningitis [LASER-TBM]
Source: Wellcome Open Res. 2021 Jun 1;6:136. [Version 1] doi: 10.12688/wellcomeopenres.16783.1 (PMC8283551; doi:10.12688/wellcomeopenres.16783.1)
Supplement: Peer review history

# Peer review: Reviewer response for version 1

**DOI:** 10.21956/wellcomeopenres.18512.r44173

**Published:** 2021-07-15

**Authors:** Frederic Ello

**Affiliations:** 1 Département de Dermatologie-Infectiologie, Unité de Formation et de Recherche des Sciences Médicales, Université Felix Houphouët-Boigny, Abidjan, Cote d'Ivoire

This is a clear and well-written protocol, for a new approach in TBM treatment. I think the aim of the study and based on strong hypotheses. Details relating to the systematic review part are clear, however I have a few changes, comments and suggestions for clarity.

1. In the abstract Page 2: 
  a. please write “tuberculosis (TB) 
  b. replace in those co-infected with HIV-1 by in patients co-infected with HIV-1.
2. Page 4: The reference does not mention the 1200 mg dose for 4 weeks. Please review the reference and suggest the correct one. Especially as this is the strategy you are proposing. One suggestion: Krzysztofiak, A., Bozzola, E., Lancella, L., Quondamcarlo, A., Gesualdo, F., & Ugazio, A. G. (2010). Linezolid Therapy of Brain Abscess. The Pediatric Infectious Disease Journal, 29(11), 1063–1064. doi: 10.1097/inf.0b013e3181f20ed5
3. Please check reference 16, the information provided does not appear in the reference in particular the data on Linezolid.
4. Page 12: add a cross for the Day 180 column in the MOCA, IHDS, EQ5d5L row, to comply with page 11 of the description in paragraph i).
5. Page 13: Below the table, add the acronyms for INR, RNA and DNA.
6. The study population consisted of all HIV-infected patients. This implies that they are either already on antiretroviral therapy or will be receiving it. As rifampicin is a potent enzyme inducer, it could cause a decrease in serum concentrations of ARVs (e.g. efavirenz or even Lopinavir…). It might be interesting to also evaluate the interactions between high doses of rifampicin, linezolid and ARVs.
7. The process of obtaining consents is not clear, especially as patients with this disease may have impaired consciousness from the outset. Otherwise added in the inclusion criteria: able to understand the content of informed consent and willing to sign the informed consent.
8. Authors should clarify what they mean by each AE will be assigned a grade 1 to 5 using the Division of AIDS (DAIDS) Table for Grading the Severity of Adult and Pediatric Adverse Events. The latest version – corrected 2.1 July 2017, and preceding ones only include
  adverse events graded to grade 1 as mild to grade 4 as life-threatening (4) but no grade 5 events.
9. It seems to me that the sample size has been calculated based on AE assumptions only, whereas the primary endpoint includes AE and death. Could you please clarify your methodological approach?

Is the study design appropriate for the research question?

Yes

Is the rationale for, and objectives of, the study clearly described?

Yes

Are sufficient details of the methods provided to allow replication by others?

Partly

Are the datasets clearly presented in a useable and accessible format?

Yes

Reviewer Expertise:

Tropicals and Infectious Diseases

I confirm that I have read this submission and believe that I have an appropriate level of expertise to confirm that it is of an acceptable scientific standard.

## References

[1] : Linezolid Therapy of Brain Abscess. *Pediatric Infectious Disease Journal* .2010;29(11) : 10.1097/INF.0b013e3181f20ed5 1063-1064 10.1097/INF.0b013e3181f20ed5 20975456

# Peer review: Reviewer response for version 1

**DOI:** 10.21956/wellcomeopenres.18512.r44287

**Published:** 2021-06-28

**Authors:** Julie Huynh

**Affiliations:** 1 Centre for Tropical Medicine, Oxford University Clinical Research Unit, Ho Chi Minh City, Vietnam

The article "Study protocol for a phase 2A trial of the safety and tolerability of increased dose rifampicin and adjunctive linezolid, with or without aspirin for HIV-associated tuberculous meningitis (LASER-TB)" is very well written. The study design is scientifically and ethically sound; data generated should inform future TBM treatment clinical trials. I have a few minor questions and comments to consider.    

 Page 4. Paragraph on Linezolid - The Li et al. study cited has important limitations which have not been highlighted. Patient selection was not consistent and transparent and the study did not report on the number of children with DR-TBM. It is therefore difficult to know if the advantage from LNZ was from treating drug-resistant TBM or TBM in general. A brief discussion of these limitations would elevate the importance of this trial in drug-susceptible TBM.

 Paragraph on HD rifampicin – In reference to the Heemskerk et al study, reviewer thinks the discussion would benefit from mentioning that although this trial did not find a mortality benefit with oral RIF 15mg/kg, nested PK studies showed survival benefit were associated with INH exposure (PMID: 31956998). ****Perhaps HD rifampicin is not the only drug we need to be increasing?

 Page 5: secondary aim: .. 'incidence of IRIS in participants stating ART'. How do authors define IRIS in the study?

 Figure 3: Authors please clarify in the legend “sparse sampling will be performed at day 3 for participants who decline intensive sampling or in whom this fails”. Are these Sparse Pk samples in addition to the day 28?

 Figure 3: it is unclear whether patients undergoing intensive PK sampling will have 4 lumbar punctures or that there are 4 timepoints at which a lumbar puncture can be  performed? Figure 1 would suggest only 1 lumbar puncture. Please clarify.

 Table 1: Solicited treatment related adverse events

 Intracerebral haemorrhage: CT or MRI finding - Will this be performed acutely where there are suggestive clinical/neurological signs/symptoms? In the absence of face-face clinic attendance, have authors considered an alternative plan  for collecting solicited treatment-related adverse events which require a physical assessment (e.g peripheral neuropathy e.t.c)? Something to consider in the current covid19 context.

 Box 2: exclusion criteria:… more than 5 days of ATT: Why was 5 days selected?

 Page 10: ..Re: gastroprotection with ranitidine. Was cimetidine, as an alternative to ranitidine, considered.  Unlike omeprazole, cimetidine does does not have drug-drug interactions with rifampicin and could simplify PK/PD analysis?

 Table 3A, 3B: it is unclear why the weight bands for oral and IV rifampicin should be different? IV rifampicin 30-33, 34-37kg, whereas PO rifampicin a single weight band covers 30-37kg. If one of the sub-study aims is 'to compare exposures between IV and oral RIF administration", should these weight bands be the same?

 Page 11 MBPNS and MTNS; are these clinical tools used widely in clinical practise at participating hospitals? If not will standardised training be provided to study staff?

 Page 11 measurements of neurocognitive function. Will assessors be blinded to drug allocation?

 Table 4: BPNS and MTNS: is there a plan to screen for neuropathy at each study visit? The table only suggests it is performed once at SCR. Haematology is missing 'X' in the table and there is an inconsistency of study timepoints for Plasma sparse sampling. The table suggests it is performed on Day 28 and Day 56 however this is different to information in figure 1.

 Page 14 MRI

 Will reporting of MRI be performed in real-time or research purposes only? If the latter, will reporting be centralized, and blinded to treatment allocation?

 Page 16 data access and handling. Will there be on-site monitoring, data validation and assessment of adherence to ICP-GCP. Please include.

Is the study design appropriate for the research question?

Yes

Is the rationale for, and objectives of, the study clearly described?

Yes

Are sufficient details of the methods provided to allow replication by others?

Yes

Are the datasets clearly presented in a useable and accessible format?

Not applicable

Reviewer Expertise:

TBM, diagnostics, pathophysiology, clinical trials

I confirm that I have read this submission and believe that I have an appropriate level of expertise to confirm that it is of an acceptable scientific standard.
